# Non-Canonical Wnt Predominates in Activated Rat Hepatic Stellate Cells, Influencing HSC Survival and Paracrine Stimulation of Kupffer Cells

**DOI:** 10.1371/journal.pone.0142794

**Published:** 2015-11-13

**Authors:** Laura Corbett, Jelena Mann, Derek A. Mann

**Affiliations:** Fibrosis Research Group, Institute of Cellular Medicine, Faculty of Medical Sciences, Newcastle University, Newcastle upon Tyne, NE2 4HH, United Kingdom; Institute of Hepatology, Foundation for Liver Research, UNITED KINGDOM

## Abstract

The Wnt system is highly complex and is comprised of canonical and non-canonical pathways leading to the activation of gene expression. Our aim was to examine changes in the expression of Wnt ligands and regulators during hepatic stellate cell (HSC) transdifferentiation and assess the relative contributions of the canonical and non-canonical Wnt pathways in fibrogenic activated HSC. The expression profile of Wnt ligands and regulators in HSC was not supportive for a major role for β-catenin-dependent canonical Wnt signalling, this verified by inability to induce Topflash reporter activity in HSC even when expressing a constitutive active β-catenin. We detected expression of Wnt5a in activated HSC which can signal via non-canonical mechanisms and showed evidence for non-canonical signalling in these cells involving phosphorylation of Dvl2 and pJNK. Stimulation of HSC or Kupffer cells with Wnt5a regulated HSC apoptosis and expression of TGF-β1 and MCP1 respectively. We were unable to confirm a role for β-catenin-dependent canonical Wnt in HSC and instead propose autocrine and paracrine functions for Wnts expressed by activated HSC via non-canonical pathways. The data warrant detailed investigation of Wnt5a in liver fibrosis.

## Introduction

Hepatic stellate cells (HSC) are widely recognised as the major cellular origin of activated pro-fibrogenic myofibroblasts in chronic liver disease, irrespective of disease aetiology. In response to liver damage HSC undergo an epigenetically-regulated transdifferentiation to adopt a myofibroblast-like phenotype characterised by proliferation, contractile ability and the secretion of vast amounts of fibril-forming extracellular matrix (ECM) proteins[1]. The persistence of these so-called activated HSC (aHSC) leads to the net deposition of ECM and the progressive remodelling of liver tissue towards a fibrotic state. Hence, aHSC are major cellular drivers of fibrogenesis and are rational targets for the design of anti-fibrotics aimed at preventing the progression of chronic liver disease to cirrhosis. Key to exploiting the aHSC for the development of anti-fibrotic strategies is a deep understanding of the regulatory cell signalling processes that dictate their fibrogenic activities.

Growing evidence suggests that tissue injury is accompanied by the reactivation of embryonic signalling pathways such as those controlled by the morphogen families Hedgehog (Hh), Notch and Wnt[2]. During development, these morphogen families are key regulators of cell fate specification, proliferation and migration indicating strong potential for a role in regulating wound repair and tissue regeneration[3,4]. Increased Hh, Notch and Wnt signalling has been implicated in promoting HSC transdifferentiation and significant improvement in fibrosis is documented in experimental models when these pathways are inhibited[5]. However, the regulation of these developmental morphogens is highly complex and knowledge of the mechanisms by which they control the phenotype and function of HSC is incomplete.

The Wnt systems is comprised of signalling proteins that are highly evolutionary conserved secreted glycoproteins with a critical role in developmental regulation[6]. In the adult, aberrant Wnt signalling has been linked to numerous pathologies, notably cancer but also bone abnormalities and arthritis[7]. Emerging evidence also supports a role in promoting tissue fibrosis in variety of organs and experimental models[8,9]. Once secreted, Wnts signal through interaction with membrane bound Frizzled (Fzd) receptors, resulting in phosphorylation of the downstream mediator Dishevelled (Dvl). Phosphorylated Dvl propagates Wnt signalling by three potential pathways: the canonical β-Catenin associated pathway, the non-canonical Planar Cell Polarity (PCP) pathway or the non-canonical Calcium associated (Wnt/Ca^2+^) pathway. The relative contributions of these distinct intracellular Wnt signalling pathways towards the regulation of myofibroblast fate and function, as well as to the control of fibrogenesis *per se*, remains poorly defined.

Recent work from a number of laboratories has suggested that Wnt signalling is activated in HSCs upon liver injury and that the expression of many components of the different Wnt pathways are modulated during HSC transdifferentiation including Wnt ligands, Frizzled receptors, the LRP5 co-receptor and the expression of nuclear β-catenin[10–13]. Moreover, the ability to modulate the fibrogenic activities of HSC with the administration of inhibitors of Wnt signalling is well documented. However, the role of canonical β-catenin-dependent Wnt signalling in HSCs is unclear due to contradictory data in the recent literature that argues for both inhibitory and activatory roles[11,13,14]. Furthermore, little attention has focussed on the potential for non-canonical Wnt signalling playing a role in HSCs. Here, we confirm using primary rodent and human cells that HSC activation is accompanied with dramatic changes in the expression of Wnt ligands and their regulatory factors. By studying the downstream intracellular Wnt regulators we fail to provide evidence for β-catenin-dependent signalling, but instead discover a role for non-canonical β-catenin-independent Wnt pathways in the control of the phenotype of aHSC and their expression of fibrogenic genes.

## Results

### Characterisation of Wnt ligands, receptors and antagonists in cultured primary HSC

**Fig 1:**
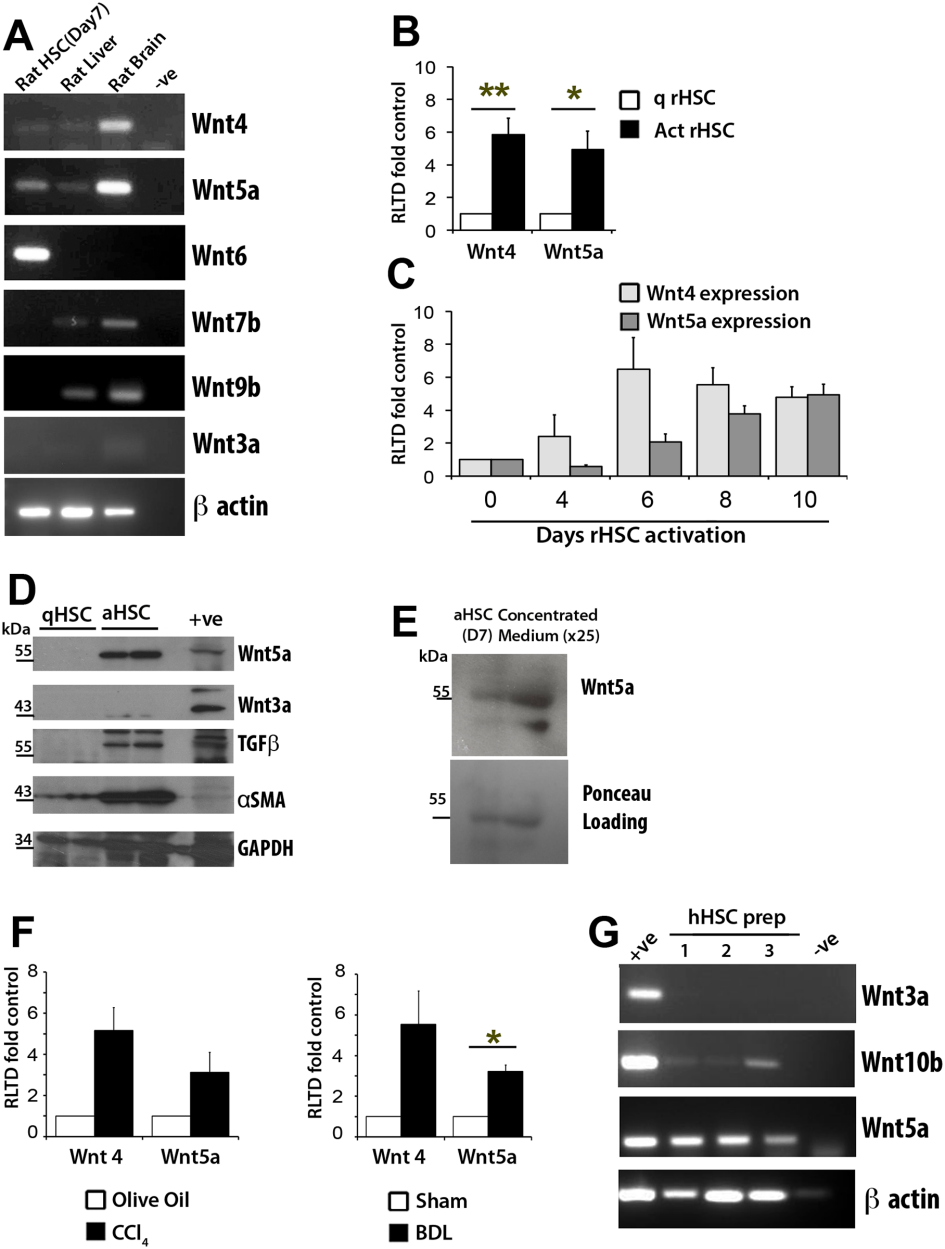
Activated HSCs express Wnt4, Wnt5a and Wnt6. (A) Products of RT-PCR for Wnt4, Wnt5a, Wnt6, Wnt7b, Wnt9b and Wnt3a in day 7 rat aHSCs, liver and brain (+ve) visualised on agarose gels using UV. Water only (-ve) serves as negative control. β-Actin serves as a loading control (B) qRT-PCR for Wnt4 and Wnt5a in culture activated rat HSCs (quiescent (qHSC) or day 7 activated HSC (aHSC) (n = 5). (C) qRT-PCR for Wnt4 and Wnt5a in rat HSCs after 0,4,6,8 and 10 days in culture, (n = 3) (D) Western Blot demonstrates increased Wnt5a expression in rat HSCs activated after 7 days in culture. Wnt3a protein expression is absent in both quiescent (qHSC) and activated (aHSC) cells. αSMA and TGFβ 1 serve as markers of HSC activation. Huh7 protein lysate serves as a positive control (E) Western Blot for Wnt5a protein in whole cell rat aHSC (Day 7) lysate and concentrated conditioned media (F) qRT-PCR for Wnt4 and Wnt5a in HSCs isolated from mouse livers injured by carbon tetrachloride injection (CCl_4_) or Bile Duct Ligation (BDL) (n = 3) (G) Products of RT-PCR for Wnt3a, Wnt10b and Wnt5a in 3 preparations of human HSCs visualised on agarose gels using UV. Wnt overexpressing LX-2 cells were used as a positive control (+ve). qRT-PCR results expressed as fold change normalised to control ± SEM. *p<0.05 (Student’s T-test).

We began by determining the expression of Wnt ligands at the transcript level in rat aHSC (Fig 1A). We observed expression of Wnt 4, 5a and 6 while Wnts 7b, 9b or 3a could not be detected. Comparison between freshly isolated qHSC and 7-day cultured aHSC demonstrated activation-associated enhancement of Wnt4 and Wnt5a indicating that these ligands may be more highly expressed in fibrogenic aHSC (Fig 1B). A more detailed time course of rat HSC activation revealed that Wnt4 and Wnt5a transcripts progressively accumulate as the cell adopts its activated phenotype with maximal expression observed between days 6 and 10 when the cells have matured to a myofibroblastic state (Fig 1C) Western blotting for alpha smooth muscle actin (α-SMA) and TGFβ confirmed that 7-day cultured rat HSC were in an activated state while blotting for Wnt5a confirmed induction of this Wnt ligand at the protein level (Fig 1D). By comparison we did not detect expression of Wnt3a protein in either qHSC or aHSC. We were also able to identify expression of Wnt5a in the aHSC conditioned media indicative of active secretion of Wnt5a protein (Fig 1E). To corroborate these data, Wnt4 and Wnt5a mRNA were induced in ex-vivo mouse HSC isolated from either CCL_4_- or BDL-injured livers (Fig 1F). Finally, expression of Wnt5a, Wnt 3a and Wnt10b was determined in human HSCs. In contrast to previous studies we failed to detect significant levels of canonical Wnt3a and only low levels of Wnt10b whereas Wnt5a was readily detectable in human HSC. (Fig 1G)

**Fig 2:**
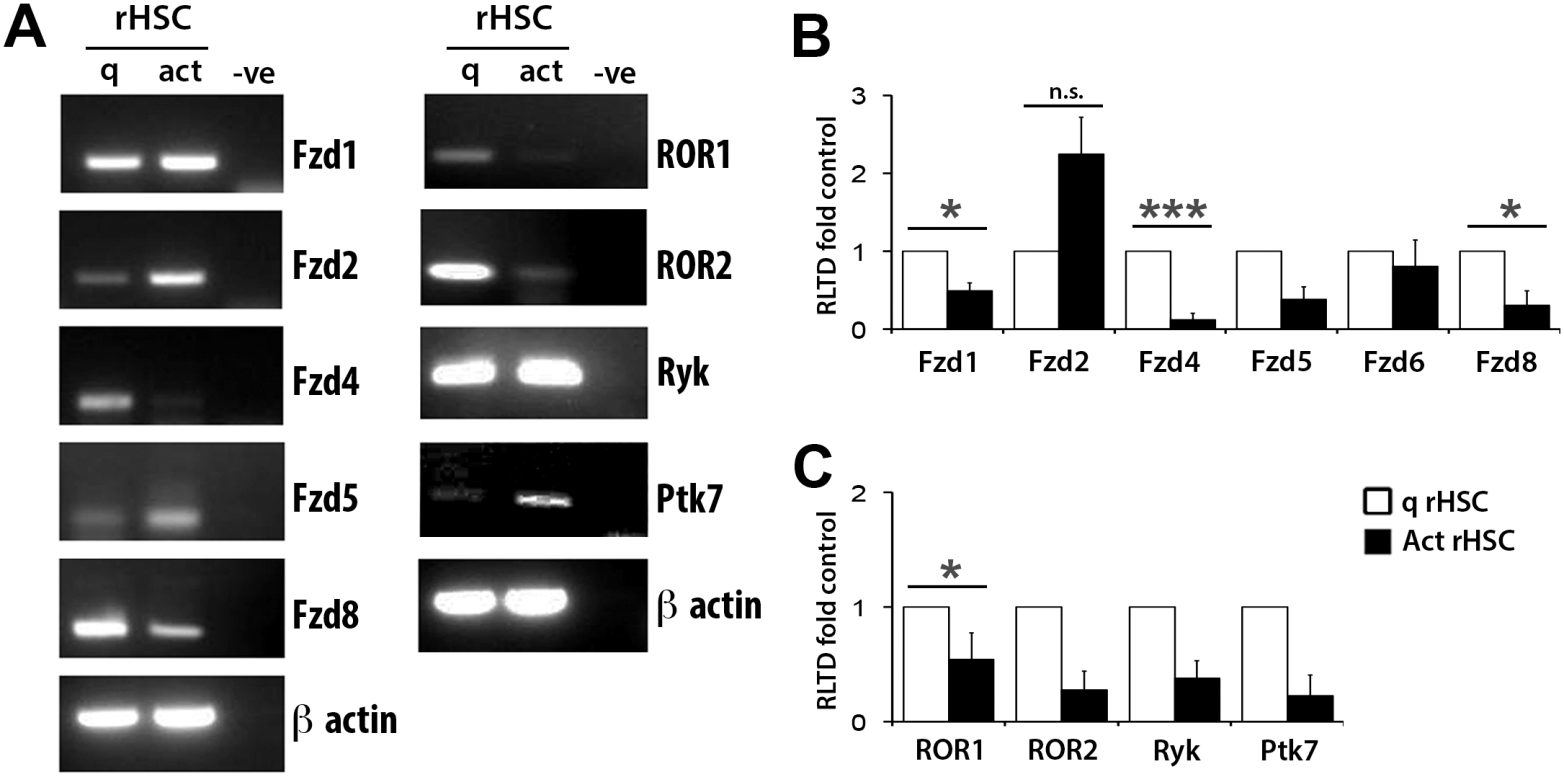
Wnt receptor expression alters upon HSC transdifferention. (A) Products of RT-PCR for Fzd Receptors and non canonical receptors in rat qHSCs and aHSCs visualised on agarose gels using UV. (B) qRT-PCR for Fzd receptors in rat qHSC and aHSC. (n = 5) (C) qRT-PCR for non-canonical receptors in rat qHSC and aHSC (n = 3), results expressed as fold change normalised to control ± SEM. *p<0.05, ***p<0.001 (Student’s T-test).

Wnt ligands trigger cell signalling via interaction with members of Frizzled (Fzd) receptors or a variety of non-canonical receptors including ROR1, ROR2, Ryk and Ptk7 [15]. Transcripts for Fzd1, Fzd2, Fzd4, Fzd5, Fzd8, ROR1, ROR2, Ryk and Ptk7 were all detected in HSC by semi-quantitative RT-PCR (Fig 2A). Comparison between qHSC and aHSC indicated that only one Fzd gene, Fzd2, is upregulated with HSC activation, whereas transcripts for Fzd1, Fzd4, Fzd8, ROR1 and ROR2 were all down-regulated in aHSC relative to qHSC. Quantitative RT-PCR confirmed these activation-associated alterations in receptor expression with enhancement of Fzd2 and suppression of Fzd4, Fzd8 and ROR1 (Fig 2B and 2C). We also confirm expression of Fzd2 and Fzd8 at the protein level. (S1A Fig)

**Fig 3:**
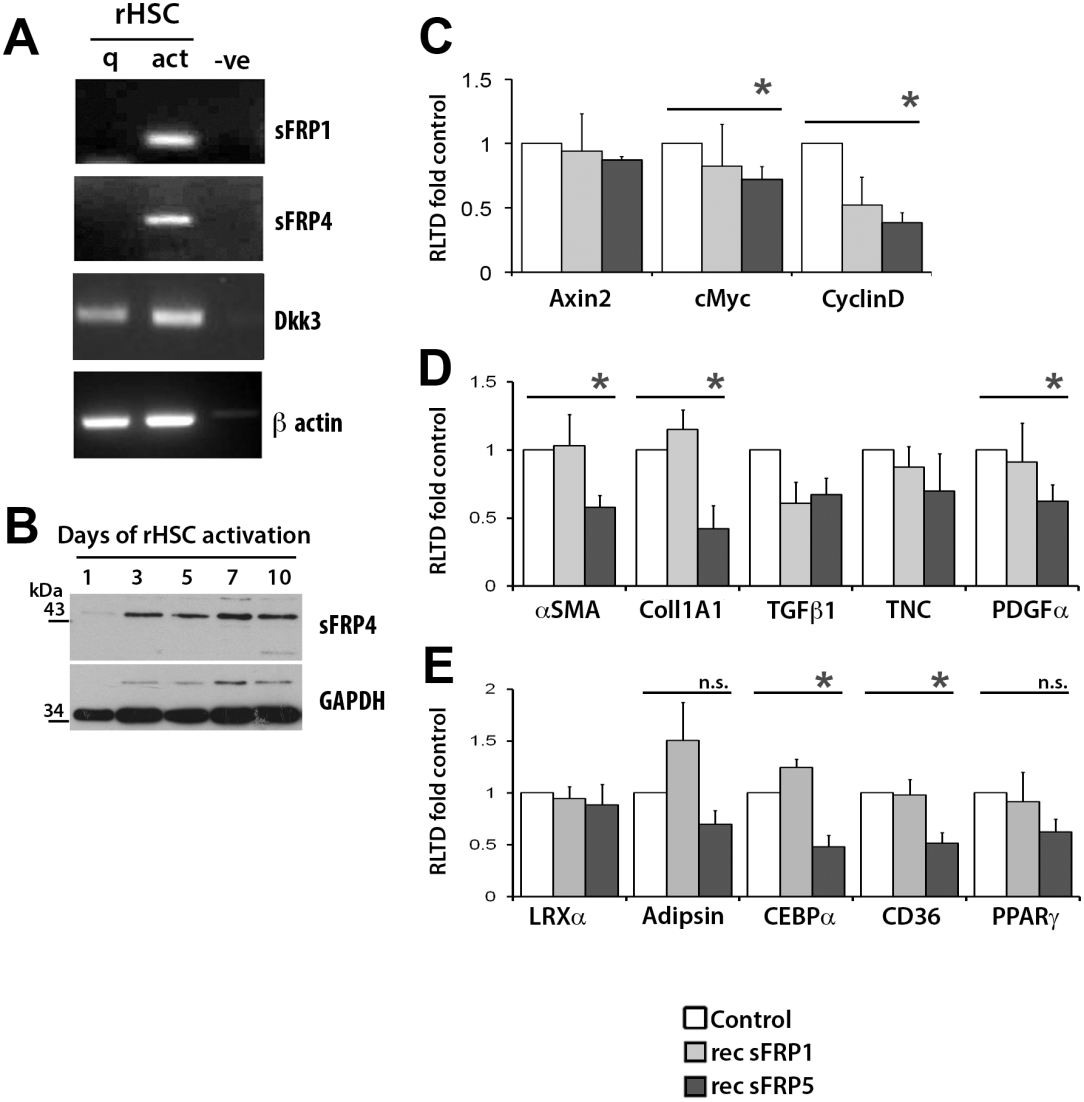
Extracellular interactors of Wnt are upregulated upon HSC transdifferentiation. (A) Products of RT-PCR for sFRP1, sFRP4 and Dkk3 in rat qHSCs and aHSCs. visualised on agarose gels using UV. (B) Western blot for sFRP4 in culture activated rat HSCs. (C) qRT-PCR for Wnt target genes in LX-2 cells treated with 5nM recombinant sFRP1 or sFRP5 (n = 4) (D) qRT-PCR for fibrogenic genes in LX-2 cells treated with 5nM recombinant sFRP1 or sFRP5 (n = 4) (E) qRT-PCR for adipogenic genes in LX-2 cells treated with 5nM recombinant sFRP1 or sFRP5 (n = 4). Results expressed as fold change normalised to control ± SEM; (n = 4). *p<0.05, (Student’s T-test).

The interaction of Wnt ligands with their cell surface receptors is under tight extracellular regulation by soluble proteins that compete for their binding; these include members of the secreted frizzled-related proteins (sFRP1-5) and the dickkopf (Dkk1-4) family. HSC activation was accompanied by induction of sFRP1, sFRP4 and Dkk3 mRNAs which was detected by both semi-quantitative (Fig 3A) and quantitative (S1B Fig) assays. By contrast only low levels of sFRP2 (S1B Fig) and other Wnt antagonists (Dkk1, Dkk2, Dkk4, sFRP5 –not shown) were found in HSC with no obvious differences in expression between quiescent and activated cells. Western blot confirmed induction of sFRP4 protein with HSC activation and maintenance of its expression in fully activated myofibroblasts (Fig 3B). Of note, canonical Wnt signalling has been reported to be critical for adipogenic differentiation and to be strongly suppressed by sFRP4 [16]. Hence, culture-induced sFRP4 (and SFRP1 and Dkk3) combined with reduced surface expression of several Wnt receptors may be important for the switch from the adipogenic phenotype of qHSC to the myofibroblastic phenotype of aHSC. Treatment of LX2 cells with recombinant sFRP1 decreased expression of the fibrogenic gene TGF1β, albeit not significantly. Treatment of LX2 cells with recombinant sFRP5 demonstrated a greater effect, significantly reducing expression of Wnt targets (cMyc, CyclinD) (Fig 3C) and fibrogenic genes (Col1A1, αSMA, PDGFα)(Fig 3D). Additionally we found a repressive effect on the expression of the adipogenesis-associated genes CEBPα and CD36, however the impact of sFRP5 on the adipogenic phenotype was limited as we found no effect on LRXα, PPARγ or adipsin (Fig 3E).

### Activated HSC do not support canonical β-catenin-dependent Wnt signalling

**Fig 4:**
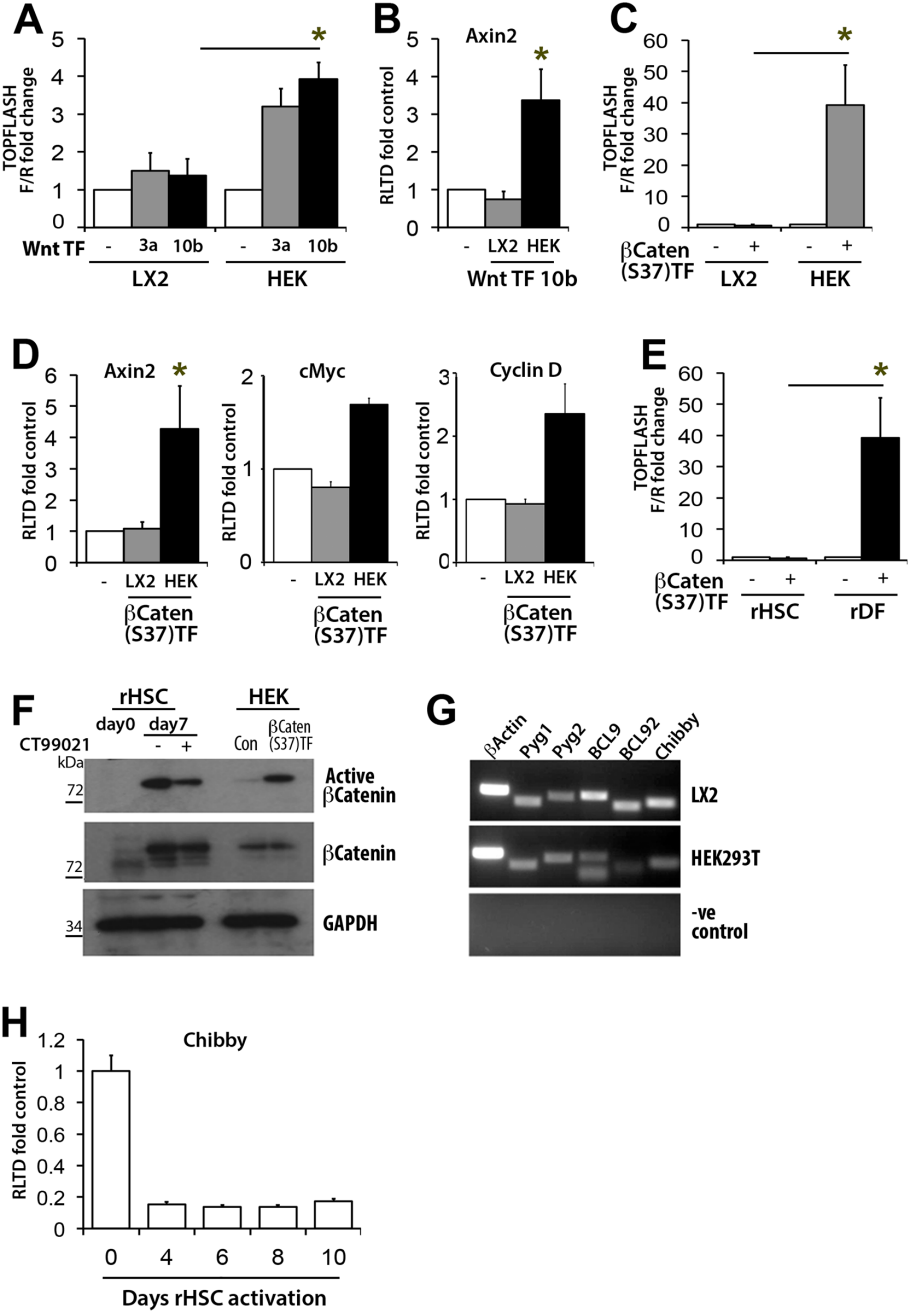
βCatenin/TCF activity appears absent in HSCs and the LX-2 cell line. (A) TOPFLASH luciferase assay in Wnt3a and 10b overexpressing HEK293 and LX-2 cells, n = 2 (B) qRT-PCR for Axin2 in Wnt10b overexpressing LX-2 and HEK293 cells n = 3 (C) TOPFLASH assay in Ser37-βCatenin overexpressing LX-2 and HEK293 cells, (n = 4) (D) qRT-PCR for Axin2, Cyclin D and cMyc in Ser37-βCatenin overexpressing LX-2 and HEK293 cells, (n = 3) (E) TOPFLASH assay in Ser37-βCatenin overexpressing rat HSCs and dermal fibroblasts (DFs), (n = 2) (F) Western Blot for total and active form of βcatenin in qHSC and aHSC treated with vehicle control or CT99021 for 24hours. Ser37-βcatenin overexpressing HEK293 cells were used as a positive control. (G) Products of RT-PCR for Pyg1, Pyg2, BCL9, BCL92 and Chibby in HEK293 and LX-2 cells visualised on agarose gels using UV. (H)qRT-PCR for Chibby in rat HSCs at 0,4,6,8 and 10 days in culture, (n = 3). Luciferase results represented as fold change of Firefly to Renilla luciferase ratio (F/R) normalised to control ± SEM. qRT-PCR results expressed as fold change normalised to control ± SEM. *p<0.05, (Student’s T-test).

**Fig 5:**
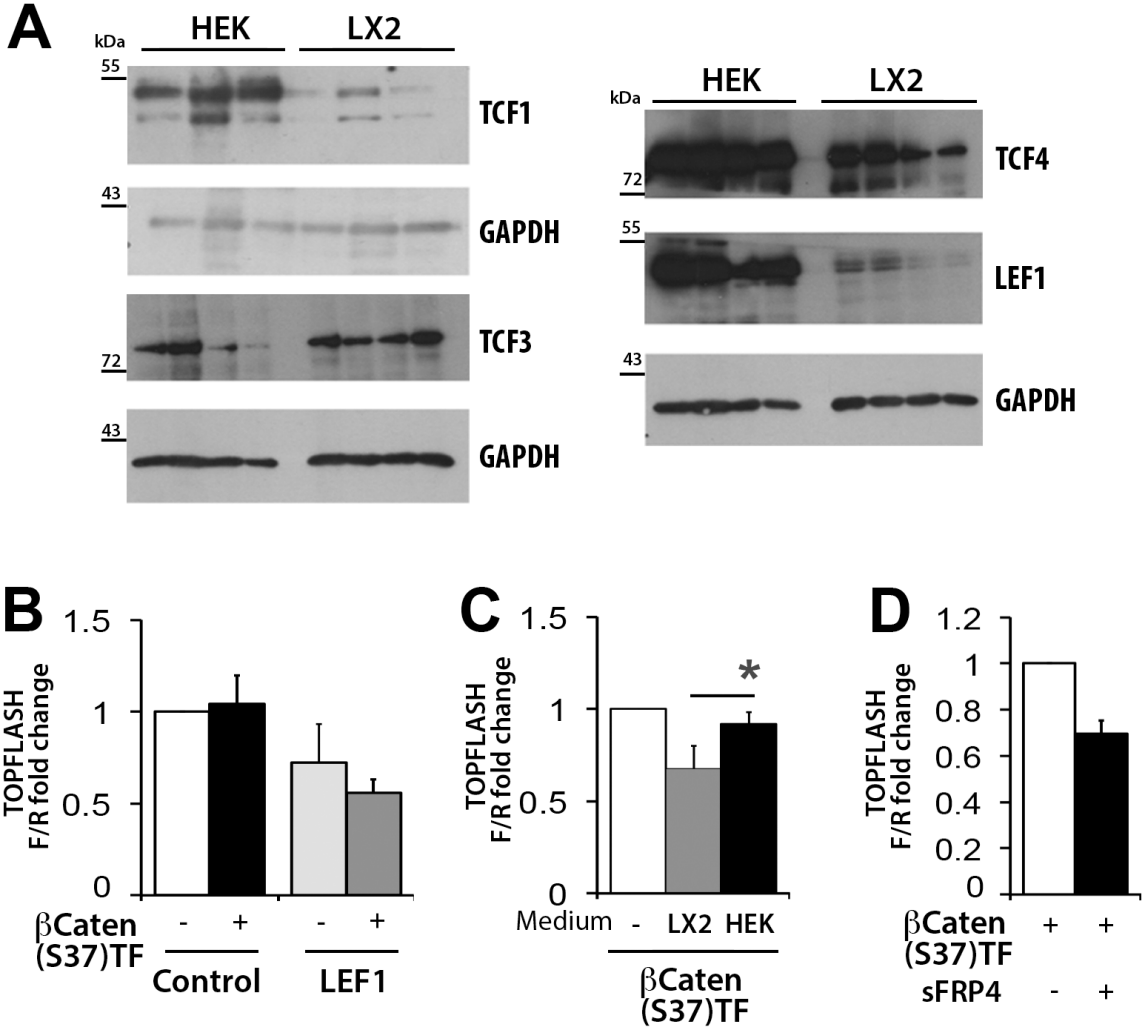
Expression of TCF/LEF transcription factors appears reduced in LX-2 cells. (A) Western Blot for TCF1, TCF3, TCF4 and LEF1in separate samples of LX-2 and HEK293 cells (B) TOPFLASH assay in HEK293 cells after 24hours incubation with conditioned medium from either HEK293 or LX-2 cells, (n = 4). (C) TOPFLASH assay in LX-2 cells overexpressing LEF1 and Ser37-βcatenin (n = 3) (D) TOPFLASH assay in HEK293 cells overexpressing Ser37-βCatenin or Ser37-βCatenin and sFRP4, (n = 3). Luciferase results represented as fold change of Firefly to Renilla *p<0.05 (Student’s *t*-test).

To determine the ability of aHSC to respond to canonical Wnt signalling, human LX-2 were co-transfected with the canonical Wnt luciferase reporter Topflash and expression vectors for Wnt3a and Wnt10b. To control these assays similar transfections were carried out in HEK293 cells which are known to support β-catenin-dependent Wnt signalling[17]. As anticipated co-transfection of the canonical Wnts activated the Topflash reporter in HEK293 cells, however no reporter activity was detected in LX-2 cells transfected with either Wnt3a or Wnt10b (Fig 4A). In addition, transfection of Wnt10b failed to induce expression of the Wnt target gene Axin2 in LX-2 which by contrast was up-regulated 3-fold by Wnt10b transfection in HEK293 cells (Fig 4B). Over-expression of a constitutive active mutant β-catenin (S2 Fig) induced Topflash activity by 40-fold in HEK293 cells (Fig 4C) and enhanced the expression of endogenous Axin2, cMyc and Cyclin D (Fig 4D). However, neither the reporter nor endogenous Axin2, cMyc and Cyclin D were stimulated in LX-2 cells, this confirming that the human HSC cell line does not support canonical Wnt signalling (Fig 4C and 4D). Expression of constitutive active β-catenin induced Topflash in primary rat dermal fibroblasts but was inactive in primary rat aHSC (Fig 4E). These data were indicative of defective β-catenin-dependent Wnt signalling in HSC. To investigate this further, rat HSC protein extracts were probed by immunoblot for expression of active (de-phosphorylated) and total β-catenin (Fig 4F) Freshly isolated qHSC lacked expression of active β-catenin, whereas HSC cultured for 7 days expressed abundant levels of active β-catenin such that pharmacological activation with a GSK3β inhibitor (CT99021) made little difference to the amount of active protein detected. Furthermore, active β-catenin was readily detected in the HSC nucleus, this ruling out a defect in nuclear translocation (S3 Fig). Once in the nucleus, β-catenin interacts with a variety of different proteins that impact on its stability and its ability to stimulate target gene transcription[18,19]. Scaffold proteins (Pyg1, Pyg2, BCL9, BCL92) that tether β-catenin to its transcription factor partners the TCF/LEF proteins, were all expressed at the mRNA level in LX-2 and HEK293 (Fig 4G) as was the nuclear repressor of β-catenin Chibby, which in primary rat HSC was found to undergo a dramatic diminution in expression with culture-induced activation (Fig 4H). The transcriptional activation of β-catenin-target genes is dependent on its interaction with TCF/LEF transcription factors [20]. Comparison of the expression of TCF/LEF proteins between HEK293 and LX-2 cells indicated a very low level of TCF1 and LEF1 in the HSC line and decreased levels of TCF4, by contrast TCF3 was expressed to a similar level between HEK293 and LX-2 (Fig 5A). These data were of particular interest since TCF1, TCF4 and LEF1 are generally considered to be activators of β-catenin-dependent transcription whereas TCF3 may have a more repressive function[21,22]. However, over-expression of LEF1 did not enable LX-2 to support transcriptional activity of co-transfected constitutive active β-catenin (Fig 5B). This result indicates that aHSC may establish multiple regulatory checkpoints to ensure blockade of canonical Wnt signalling. Indeed, exposure of HEK293 cells to LX-2-conditioned media is able to partly inhibit constitutive active β-catenin signalling (Fig 5C), which may be due to the secretion of inhibitory sFRPs by HSC, this idea being confirmed by demonstrating the ability of overexpressed sFRP4 to inhibit Topflash reporter in HEK293 transfected with constitutive active β-catenin (Fig 5D).

### Non-canonical Wnt signalling in HSCs

**Fig 6:**
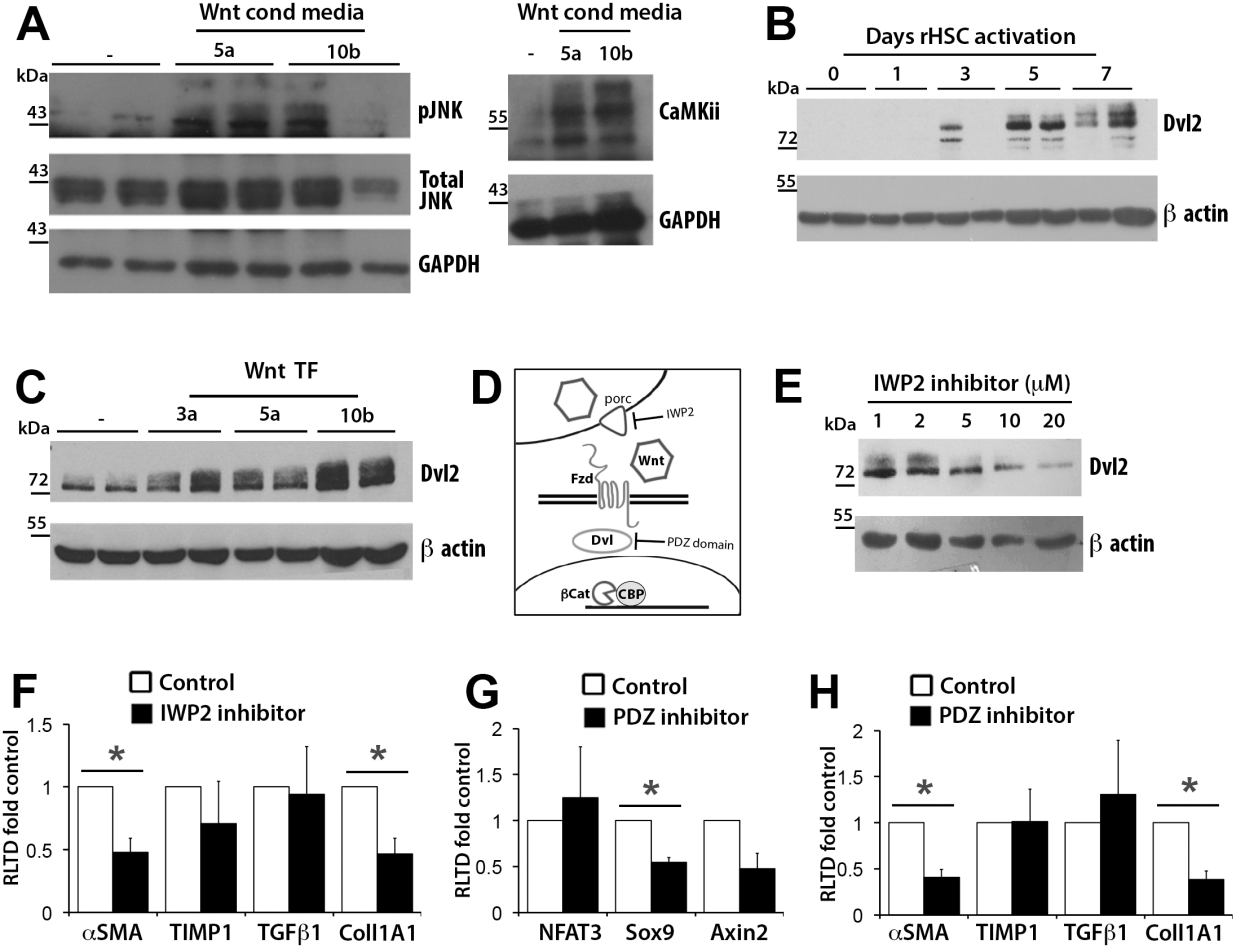
HSCs upregulated non-canonical effectors upon Wnt stimulus. (A) Western blot for total (T) and phosphorylated (P) forms of JNK and CamKii in two preparations of rat aHSCs treated for 24 hours with either control (-), Wnt5a or Wnt10b conditioned media. (B) Western blot for Dvl2 in two preparations of rat HSCs at 0,4,6,8 and 10 days in culture. (C) Western Blot for Dvl2 in LX-2 cells overexpressing Wnt3a, Wnt5a or Wnt10b. (D) Diagram illustrating points in Wnt pathway for which inhibitors were chosen: Wnt secretion (IWP2) and Dvl-PDZ domain function (Dvl-PDZ). (E) Western Blot for Dvl2 in rat HSCs treated with 1–20 μM of IWP2 (F) qRT-PCR for profibrotic markers in vehicle control (DMSO) and 20μM IWP2 treated rat aHSCs, (n = 4). (G)qRT-PCR for NFAT3, Sox9 and Axin2 in vehicle control (DMSO) and 5μM PDZ-Inhibitor treated rat aHSCs, (n = 3) (H) qRT-PCR for profibrotic markers in vehicle control and 5μM PDZ-Inhibitor treated rat aHSCs (n = 3). qRT-PCR results expressed as fold change normalised to control ± SEM *p<0.05 (Student’s *t*-test).

Having failed to find evidence for β-catenin-dependent canonical Wnt activity in HSC we turned our attention to non-canonical Wnt signalling which is mediated via JNK- and CamKii/NFAT-dependent pathways[23,24]. Exposure of primary rat HSC to Wnt ligands Wnt5a and Wnt10b led to a substantial induction of active phosphorylated JNK (p-JNK) and enhanced expression of CamKii (Fig 6A). Dishevelled proteins (Dvl1-3) function as key membrane-proximal intracellular mediators of canonical and non-canonical Wnt signalling [25]. Phosphorylation of Dvl2 has been functionally associated with activation of non-canonical Wnt signal transduction and can be assessed by Dvl2 electrophoretic mobility shift[26]. Immunoblotting for Dvl2 with protein extracts from primary rat HSC representing a time course for culture-induced activation not only showed activation-dependent induction of Dvl2 expression (appearing at culture day 3) but also revealed a progressive electromobility shift as a function of days in culture, with day 7 aHSC predominantly expressing the low mobility hyperphosphorylated modified Dvl2 (Fig 6B). Of note, treatment of protein extracts with alkaline phosphatase selectively depleted this lower mobility form of Dvl2 confirming that the electrophoretic shift is due to phosphorylation (S4 Fig) Over-expression of Wnt3a, Wnt5a or Wnt10b into LX-2 cells induced an Dvl2 electrophoretic mobility shift confirming the ability of HSC to support Wnt signalling via Dvl2 activation (Fig 6C). Moreover, media collected from Wnt5a transfected LX-2 (S5 Fig) was able to activate low-mobility forms of Dvl2 in primary rat HSC (S6A Fig) and stimulated a modest increase in expression of α-SMA and transforming growth factor beta (TGFβ1) transcripts (S6B Fig). To determine the functional relevance of Wnt signal transduction in HSC the effects of Wnt inhibitors that have distinct molecular targets (as illustrated in Fig 6D) was examined in primary rat aHSC. IWP2 is an antagonist of Porcupine (Porc), which is responsible for the palmitoylation and subsequent secretion of Wnt ligands[27]. Dvl-PDZ targets the central PDZ domain of the Dvl proteins preventing their interaction with Fzd receptors[28]. ICG001 is an inhibitor of β-catenin interaction with its transcriptional co-activator CBP and as such is an inhibitor of canonical Wnt signalling[29]. Treatment of rat aHSC with IWP2 dose-dependently suppressed p-Dvl2 (top band on Western blot, Fig 6E) and suppressed α-SMA and collagen IA1 mRNA expression (Fig 6F). Dvl-PDZ, which is expected to suppress all downstream Wnt signalling, lowered expression of common Wnt target genes Sox9 and Axin2 (Fig 6G) and (as seen with IWP2) also significantly inhibited expression of α-SMA and collagen IA1 transcripts (Fig 6H). By contrast ICG001 was without effect on fibrogenic gene expression (S7A Fig) but did appear to cause rounding up of cells and apoptosis as determined by acridine orange staining (S7B and S7C Fig). We conclude from the effects of IWP2 and Dvl-PDZ that non-canonical Wnt signalling contributes to regulation of fibrogenic gene expression in aHSC.

### Wnt5a protects HSC from apoptosis and stimulates fibrogenic characteristics of Kupffer cells

**Fig 7:**
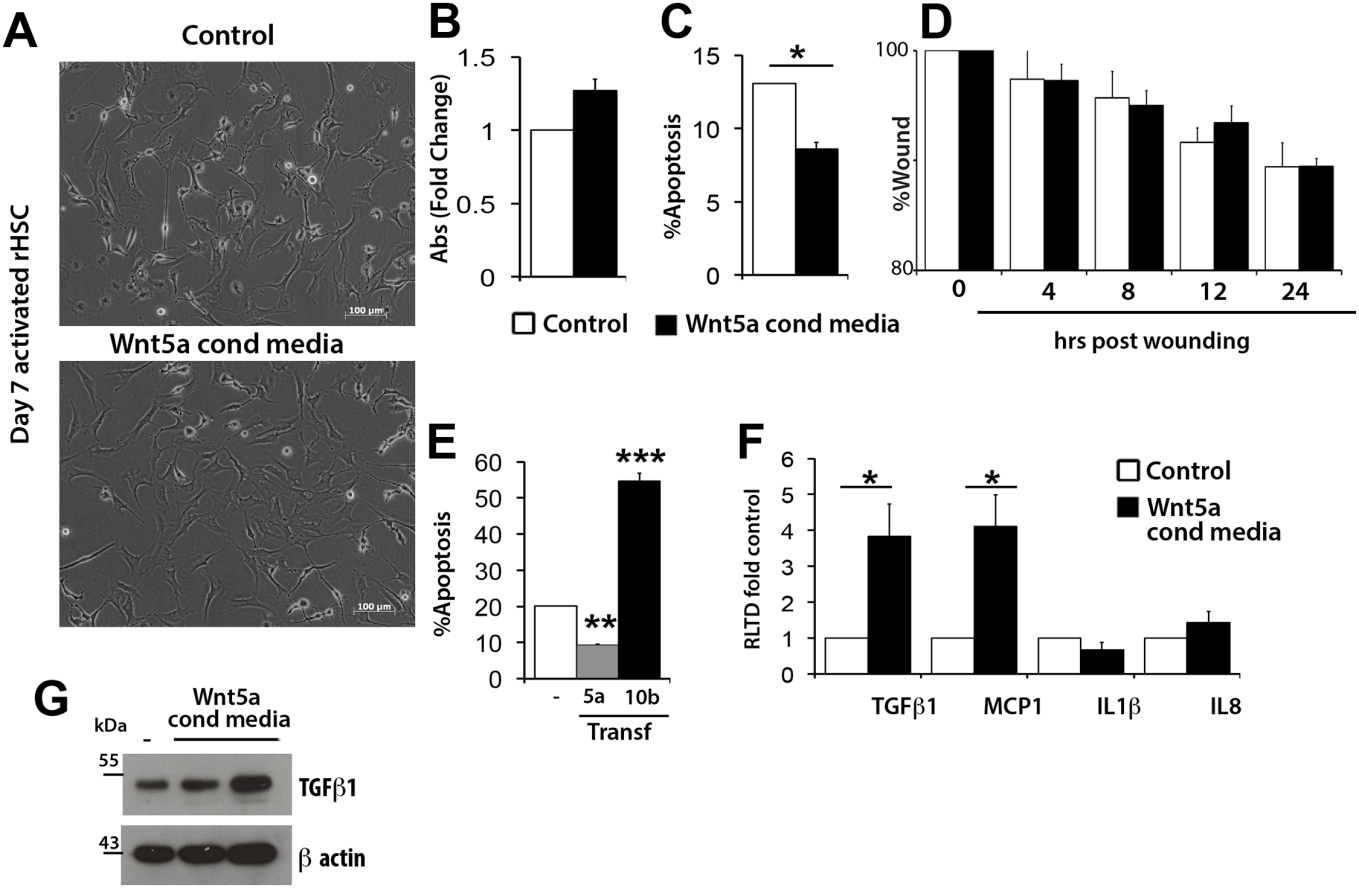
Wnt5a stimulus influences HSC survival and expression of profibrotic markers in Kuppfer cells. (A) Rat aHSCs were treated with control or Wnt5a conditioned medium and visualised by bright field microscopy. (B) Proliferation was assessed by MTT assay (C) Acridine Orange staining was used to quantify apoptotic response upon serum withdrawal (D) Migratory potential was assessed by scratch wound assay (E) Apoptotic response in LX-2 cells overexpressing Wnt 5a orWnt10b was also assessed by acridine orange staining in standard culture conditions (10% FCS) or serum free conditions. Data presented as number of apoptotic cells as percentage of total cell number. (F) qRT-PCR for profibrotic markers in control or Wnt5a conditioned medium treated rat Kuppfer cells, (n = 3) (G) Western Blot for TGFB1 expression in control or Wnt5a conditioned medium treated Kuppfer cells. qRT-PCR results are expressed as fold change normalised to control ± SEM *p<0.05, **p<0.01, ***p<0.001 (Student’s *t*-test).

Our observation that HSC secrete and respond to Wnt5a by activating Dvl2 and downstream non-canonical Wnt signalling encouraged us to further examine the functional contribution of this Wnt ligand to fibrogenesis. As before, primary rat aHSC were exposed to conditioned media from cultures of Wnt5a over-expressing LX-2 cells prior to determining effects on morphology (Fig 7A), proliferation by MTT assay (Fig 7B) and expression of PCNA and c-Myc (S8 Fig), apoptosis by acridine orange staining (Fig 7C and S9 Fig) and wound-induced migration by scratch wound assay (Fig 7D). The only phenotypic characteristic that was responsive to Wnt5a was HSC apoptosis where a modest protection to serum-starvation-induced cell death was measured (Fig 7C). We were also able to show protective effects of Wnt5a in transfected LX-2, whereas by contrast over-expression of Wnt10b appeared to stimulate apoptosis (Fig 7E). Finally, we were interested to determine if HSC-derived Wnt5a is able to exert paracrine effects on Kupffer cells (KC). To this end, primary rat KC were exposed to either control LX-2 conditioned media or to media conditioned by LX-2 transfected with Wnt5a. Measurement of transcripts for secreted inflammatory and fibrogenic mediators was performed and indicated that paracrine Wnt5a can stimulate KC expression of pro-fibrogenic TGFβ1 and monocyte chemoattractant protein 1 (MCP1) (Fig 7F). Western blot confirmed that Wnt5a induced TGFβ1 at the protein level (Fig 7G). We conclude that Wnt5a can exert autocrine control over HSC survival and stimulate the expression of soluble profibrogenic mediators by KC, this suggests that Wnt5a contributes to the persistence of fibrogenesis in the diseased liver.

## Discussion

The Wnt signalling system has been the subject of considerable attention in HSCs and liver fibrosis with recent reports suggesting functions in HSC transdifferentiation, proliferation, apoptosis, control of fibrogenic gene expression and regulatory interactions of aHSC with other liver cell types and physiological responses such as hepatocellular regeneration[11,13,14]. However, a clear understanding of which components of the highly complex Wnt system are of functional importance in HSC is lacking, as is knowledge of signalling pathways through which Wnt ligands mediate their fibrogenic effects. In particular, the literature is confusing concerning the role played by canonical β-catenin-dependent Wnt signalling, with some reports claiming active canonical Wnt regulating functions of aHSC, but others suggesting that canonical Wnt operates in qHSC and is suppressed in aHSC[11,13,14]. We therefore considered it timely to carry out a detailed analysis of the expression of Wnt ligands, receptors and regulators in HSC and to ask with primary HSC which Wnt signalling pathways are active and contributing physiologically to HSC function and fibrogenesis.

**Fig 8:**
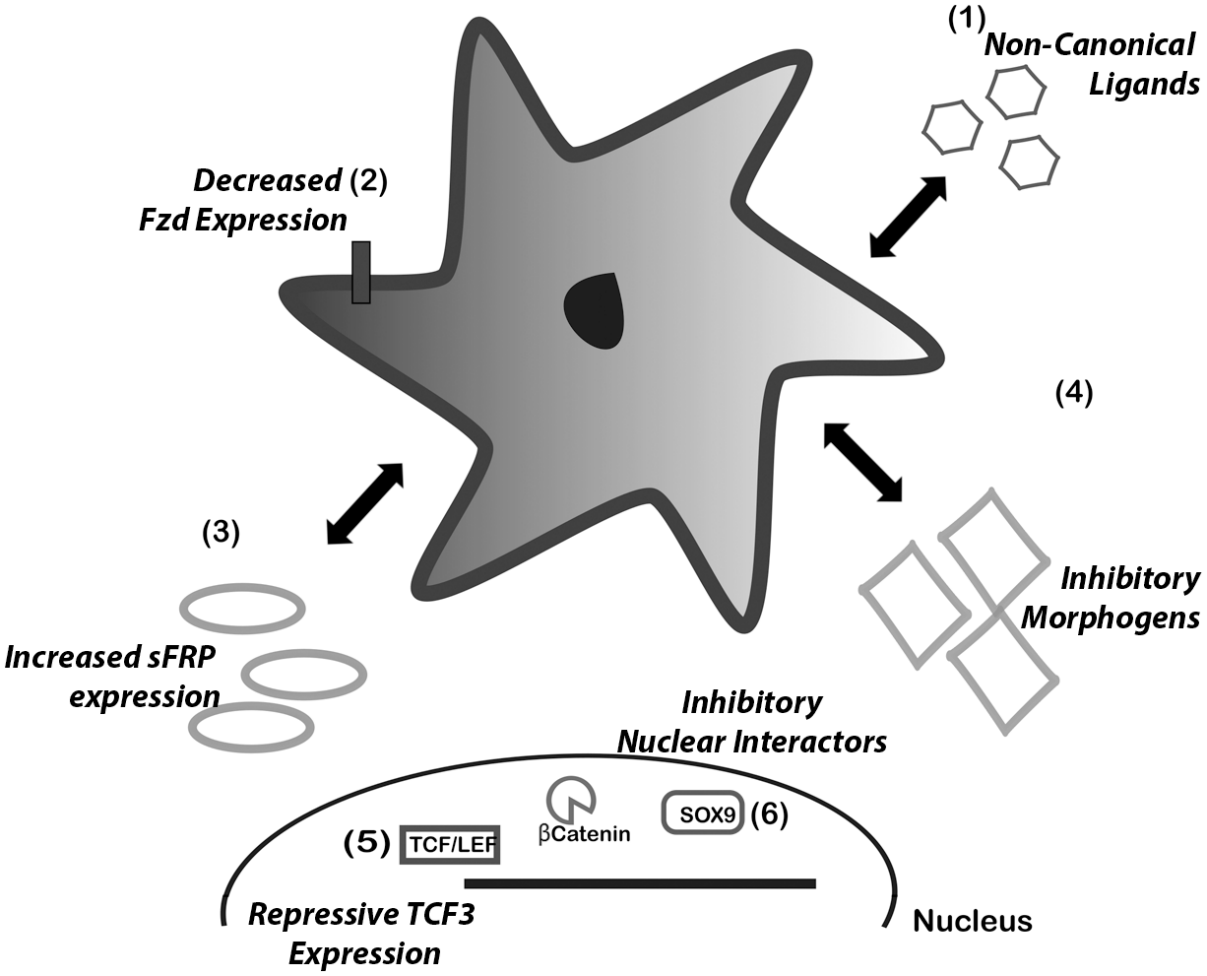
Possible Points of Canonical Wnt Inhibition. This study has highlighted six possible points of canonical Wnt inhibition occurring in HSCs. (1) Predominantly non-canonical ligand expression. Non canonical signalling is inhibitory to canonical signalling (2) Decreased Fzd receptor expression, reducing response to canonical ligands secreted by other cells (3) Increased sFRP expression (4) Increased expression of other morphogens such as Notch, able to directly inhibit βCatenin. In the nucleus, HSCs express higher levels of the repressive TCF3 in comparision to the other TCF/LEF family members associated with transcriptional activation(5) (6) Transdifferentiation is associated with upregulation of SOX9, direct inhibitor of βCatenin activity.

The main conclusion from our data is that at least *in vitro* there is little concrete evidence in favour of a role for canonical β-catenin-dependent Wnt signalling in aHSC. Rather on the contrary, the aHSC is lacking a number of factors for this pathway to be active (Fig 8). These deficiencies include low-level autocrine production of canonical Wnt ligands, a global down-regulation of Fzd receptor genes, abundant expression of repressive sFRP proteins including sFRP4, a well-established suppressor of β-catenin-dependent signalling[16], and low-level expression of the transcriptional mediators TCF1, TCF4 and LEF1. Despite repeated attempts in LX-2 and with numerous independent cultures of primary HSC we were unable to obtain measurable levels of β-catenin-dependent Topflash activity. Surprisingly this was also the case when co-transfecting with a constitutive active β-catenin that bypasses the need for upstream signalling events. The precise deficiency in aHSC that prevents β-catenin-dependent Wnt signalling is not yet clear but may as we suggest be due to a combined low-level expression of numerous key regulatory components. Alternatively, we have shown that as HSC transdifferentiate they acquire abundant expression of hyper-phosphorylated Dvl2, which in this form negatively regulates canonical Wnt[30]. Conceptually it is not unreasonable for canonical Wnt signalling to be suppressed in aHSC given that Wnt-activated β-catenin is required for adipogenesis[31]. Highly active canonical Wnt signalling may be incompatible with HSC transdifferention and as previously reported by Kordes *et al* [13] is more expected to be a feature of the adipogeneic phenotype of the qHSC. Despite these observations we were able to confirm the expression of β-catenin in aHSC and its localisation to the nucleus. A likely explanation for this apparent paradox is that β-catenin might function as a signalling molecule outside of the context of the canonical Wnt pathway and in this way be required for aHSC phenotype and/or function. As an example, interactions between β-catenin and members of the TGF-β1 regulated Smad proteins have been observed indicating coupling of Wnt and Smad components that may expand the mechanisms by which these proteins regulate gene transcription[32,33]. In order to properly define functions for β-catenin in HSC and their fibrogenic activities it will be necessary to genetically manipulate the β-catenin gene *in vivo* and we await these future experiments with much interest.

Our second conclusion was that aHSC abundantly express and secrete Wnt5a, which may through non-canonical signalling pathways and via both autocrine and paracrine mechanisms promote fibrogenesis. In previous work Wnt5a was reported as an induced transcript with culture-activation of rat HSC[11,12] and by proteomic analysis of LX-2 to be a component of the fibrotic ECM [14]. Here, we confirm Wnt5a expression by aHSC and extend these reports by demonstrating active secretion of the ligand by aHSC, autocrine suppression of HSC apoptosis and paracrine stimulation of TGF-β1 and MCP-1 fibrogenic factors by KC. Rashid *et al* were able to localise Wnt5a to fibrotic and peri-fibrotic regions of diseased human and rodent liver tissue suggesting that aHSC may be a predominant source of this Wnt ligand in the diseased liver (15). Studies in lung fibroblasts have implicated a role for Wnt5a as a regulator of proliferation and apoptosis, hence our finding for an anti-apoptotic role for Wnt5a in aHSC may be indicative of this being a core function for Wnt5a in tissue fibrosis[34]. The mechanism(s) by which Wnt5a promotes aHSC survival remain to be determined, however we would argue for a β-catenin-independent mechanism. Of relevance, Wnt5a can suppress apoptosis in other cell types via activation of NF-κB- and/or NFAT-dependent signalling pathways[35,36]. Our laboratory and other investigators have established a critical aHSC survival function for NF-κB, it will therefore be of interest to investigate the degree to which the elevated constitutive NF-κB activity of aHSC is influenced by Wnt5a[37]. Furthermore, in macrophages NF-κB and Wnt5a establish a self-sustaining regulatory circuit that supports macrophage survival[38]. The role of KC as potentiators of HSC transdifferentiation is well established, in particular KC-derived TGF-β1 is required for initiating this process. However, there is increasing awareness that communication between HSC and hepatic immune cells is a two-way process with potential for HSC to modulate the behaviour of macrophages, T cells and neutrophils via secreted mediators. We were able to show that media conditioned by Wnt5a-transfected HSC stimulates elevated expression of a subset of KC genes including TGF-β1 and MCP1. Both of these KC-derived cytokines have stimulatory effects on HSC including collagen synthesis and migration[39] indicating that Wnt5a contributes to complex two-way paracrine pro-fibrogenic signalling networks established between aHSC and KC. Determining the degree to which HSC-derived Wnt5a is required to maintain these intercellular signalling networks and its functional contribution to liver fibrosis will be important to discover but will require genetic studies in which Wnt5a expression is selectively deleted in HSC.

In conclusion, the Wnt signalling system is a functional feature of HSC-derived myofibroblasts and has the potential to influence fibrogenesis via autocrine and paracrine routes. However, from our data it seems that the degree to which aHSC utilise the canonical Wnt pathway is minimal and as such it will be important in future work to interrogate the nature and function of non-canonical Wnt signalling.

## Materials and Methods

### Cell Isolation and Cell Culture

All experiments were approved by Newcastle University’s Animal Welfare and Ethical Review Board. Animals were bred in the Newcastle University animal unit, maintained as specific pathogen free according to the FELASA Guidelines. Work was carried out under project and personal licences approved by the UK Home Office. HSCs were isolated from normal livers of male Sprague-Dawley rats (following Ketamine/Xylazine anaesthesia and sacrifice by exsanguination) by collagenase/pronase perfusion by density centrifugation in Optiprep (Invitrogen). Isolated cells were pre-plated on plastic flasks for 20 mins to allow attachment and separation of Kupffer cells. Cells harvested directly after isolation (Day 0 of culture) were considered quiescent. Cells harvested after 7 days of culture were considered activated. Dermal fibroblasts were obtained from freshly harvested rat skin by collagenase A digestion. Primary cells were maintained in humidified atmosphere of 5% CO_2_ in air at 37°C, cultured in Dulbecco’s modified Eagle’s medium (DMEM) supplemented with 16% foetal bovine serum (FBS), 100 U/mL penicillin, 100 g/mL streptomycin and 2mM L-glutamine. The LX-2 cell line, a spontaneously immortalized human hepatic stellate cell, was kindly provided by Dr. Scott L. Friedman (Mount Sinai School of Medicine, New York, NY). Treatment reagents used were recombinant sFRP1 and sFRP5 (5nM) (R&D systems), CT99021 (Axon MedChem), IWP2 (20μM) (Sigma), Dvl-PDZ Domain inhibitior (Calbiochem) (5μM), ICG001 (5μM)(Tocris). Amicon Ultra-0.5 mL centrifugal filters (Millipore) were used to concentrate cell culture medium for western blotting.

### RNA extraction, cDNA synthesis and quantitative reverse transcriptase-polymerase chain reaction (RT-PCR)

Total RNA was purified using the RNeasy kit (Qiagen) and cDNA templates prepared using a random hexamer primer and Moloney murine leukemia virus reverse transcriptase (MMLV-RT) (Promega). RNA from HSCs isolated from BDL or CCl_4_ injured mouse liver was donated by H. Tsukamoto, as used in Mann *et al* [40]. RNA from human HSCs isolated from normal margins of livers resected for removal of metastatic colorectal tumors was donated by F.Oakley, as used in Ebrahimkhani et al [41]. PCR primers were designed using Oligo Primer Analysis Software 4.0 (Molecular Biology Insights, Inc) (S1 Table). Reverse Transcriptase PCR (RT-PCR) was conducted using PCR master mix (Promega) following a 35 cycle program. Quantitative polymerase chain reaction (qPCR) was performed using a 7500 Fast system (Applied Biosciences) following a 40 cycle program. All results were normalised to a control housekeeping gene: GAPDH (Human) or β-Actin (Rat). Melt curve analysis was employed to confirm presence of a single PCR product. The relative level of transcriptional difference calculated by using the following equation: [1/(2A)] ×100.

### SDS-PAGE and Immunoblotting

Sodium dodecyl sulfate–polyacrylamide gel electrophoresis (SDS-PAGE) and immunoblotting were done as previously described. Primary Antibodies used were CaMKII (pan) (#3362S), Dvl2 (#3216), LEF1(C12A5)(#2230), PJNK (Thr183/Tyr185) (#9251), TCF1 (C63D9) (#2203), TCF3 (D15G11) (#2883), TCF4 (C48H11) (#2569), TGFβ (56E4) (#3709), TJNK (#9252), Wnt5a (C27E8) (#2530) and αTubulin (#2144) (1 in 1000) (Cell Signal), Wnt5a (#55184-1-AP), Wnt3a (#21414-1-AP), Fzd2 (#24272-1-AP) (1 in 1000), sFRP4 (#15328-1-AP) (1 in 500) (Proteinech). GAPDH (ab9485), β-Actin(AC-15) (ab6276), β-Catenin (Total) (ab6302), Fzd8 (ab75235) (1 in 2000) (Abcam), p84 (5E10) (GT70220) 1 in 1000, (Genetex), β-Catenin(Active) (8E7) (#05–665) 1 in 500 (Millipore).

### Migration Assay

Cells in 6 well plates were pre-treated with Mitomycin-C (Sigma) for 30 minutes and single scratch made in each well. Wound was measured over a 24 hour period. Results presented are averages of ten measurements per field. Experiments were performed in triplicate.

### Acridine Orange

Cells were seeded in 12-well plates at 20–30% confluency. Apoptotic cells were detected by Acridine Orange staining (1μg/ml) (Sigma) and visualisation under a FITC filter. Five random fields per well were counted at x20 magnification in duplicate wells.

### MTT Assay

Cells seeded in 96 well plates were treated with MTT (5mg/ml, Sigma) for 4 hours. Medium was then aspirated and cells incubated with DMSO (Sigma) for 30 minutes. Plate was read using plate reader at 570nm absorbance.

### Transfections and reporter gene assays

Cells were transfected with 1μg of plasmid DNA using Effectene transfection reagent (Qiagen) for 48 hours before harvest. For TOPFLASH reporter assay, 0.5μg of TOPFLASH plasmid (Millipore) was contransfected with 0.05μg of renilla luciferase plasmid serving as a transfection control. Luminescence was measured using the Dual Luciferase Reporter Kit (Promega) and data presented as average ratios of firefly to renilla luciferase activity. Conditioned medium was collected from transiently transfected LX-2 cells over expressing Wnt5a, Wnt10b or an empty control construct.

### Constructs

Active β-catenin construct (S37A) was provided by D.L.Johnson, as used in Orford *et al*[42]. Wnt3a, Wnt5a, Wnt10b, LEF1 and sFRP4 constructs were sourced from Origene.

### Statistical Analysis

Data are expressed as mean ± standard error of the mean (SEM). P values were calculated using a two-tailed paired or unpaired Student t test. For figures with statistically significant data *, **, and *** indicate P values of <0.05, <0.01 and <0.001, respectively.

## Supporting Information

S1 Fig (A) Western Blot analysis confirms protein expression of Fzd2 and FZd8 in qHSC and aHSC. Fzd overexpessing LX2 cells serve as a positive control. (B) qRT-PCR analysis of sFRPs and Dkk 3 in rat qHSC and aHSC. Results expressed as fold change normalised to control ± SEM (n = 4).
(TIF)

S2 Fig Western Blot confirming increased protein expression of active form of βCatenin upon overexpression of S37 mutant βCatenin in LX2 and HEK293 cells. (TIF)

S3 Fig Western Blot confirming presence of βCatenin in both nuclear and cytoplasmic fractions of LX2 cells. Nuclear βcatenin is increased upon overexpression of S37 mutant. αTubulin and p84 serve as controls of cytoplasmic and nuclear purity respectively.
(TIF)

S4 Fig Western Blot demonstrating reduced expression of hyperphosphorylated form of Dvl2 upon treatment of protein lysates with the dephosphorylating agent Alkaline Phosphatase (ALP). (TIF)

S5 Fig Western Blot demonstrating presence of Wnt5a protein in conditioned media collected from Wnt5a overexpressing LX2 cells. Complete media (16% FBS containing DMEM) serves as a negative control.
(TIF)

S6 Fig (A) Western Blot demonstrating increased expression of Dvl2 in day 7 rat HSCs upon Wnt5a or Wnt10b conditioned medium treatement (B) qRT-PCR for profibrotic markers in rat HSCs treated with Wnt5a conditioned medium at Day3 or Day 7 of in vitro culture. Results expressed as fold change normalised to control ± SEM (n = 3).
(TIF)

S7 Fig (A) mRNA levels of profibrotic markers were analysed by qPCR. Results expressed as fold change normalised to control ± SEM; n = 3 (B) Bright field microscopy images (C) Apoptotic response analysed by Acridine Orange staining. Data presented as number of apoptotic cells as percentage of total cell number (n = 3).
(TIF)

S8 Fig Western blot demonstrating expression of proliferation markers PCNA and cMyc in Wnt5a treated rat HSCs. (TIF)

S9 Fig Acridine Orange staining demonstrating increased number of apoptotic cells (arrows) in control cells compared to Wnt5a overexpressing cells after serum starvation. (TIF)

S1 Table Quantitative PCR primers used for cDNA amplification. (DOCX)

## References

[1] Friedman SL (2008) Mechanisms of Hepatic Fibrogenesis. Gastroenterology 134: 1655–1669. 10.1053/j.gastro.2008.03.003 18471545 PMC2888539

[2] Selman M, Pardo A, Kaminski N (2008) Idiopathic Pulmonary Fibrosis: Aberrant Recapitulation of Developmental Programs? PLoS Med 5: e62 10.1371/journal.pmed.0050062 18318599 PMC2265304

[3] Beyer C, Dees C, Distler JHW (2013) Morphogen pathways as molecular targets for the treatment of fibrosis in systemic sclerosis. Archives of Dermatological Research 305: 1–8. 10.1007/s00403-012-1304-7 23208311

[4] Tsukamoto H, Zhu NL, Wang J, Asahina K, Machida K (2012) Morphogens and hepatic stellate cell fate regulation in chronic liver disease. Journal of Gastroenterology and Hepatology (Australia) 27: 94–98. 10.1111/j.1440-1746.2011.07022.x PMC3337168 22320925

[5] Chen Y, Choi SS, Michelotti GA, Chan IS, Swiderska-Syn M, Karaca GF, et al (2012) Hedgehog controls hepatic stellate cell fate by regulating metabolism. Gastroenterology 143: 1319–1329.e1311 10.1053/j.gastro.2012.07.115 22885334 PMC3480563

[6] Logan CY, Nusse R (2004) The Wnt signaling pathway in development and disease. Annual Review of Cell and Developmental Biology. pp. 781–810. 15473860 10.1146/annurev.cellbio.20.010403.113126

[7] Cadigan KM, Peifer M (2009) Wnt Signaling from Development to Disease: Insights from Model Systems. Cold Spring Harbor Perspectives in Biology 1. 10.1101/cshperspect.a002881 PMC2742092 20066091

[8] Akhmetshina A, Palumbo K, Dees C, Bergmann C, Venalis P, Zerr P, et al (2012) Activation of canonical Wnt signalling is required for TGF-β-mediated fibrosis. Nat Commun 3: 735 10.1038/ncomms1734 22415826 PMC3316881

[9] Beyer C, Schramm A, Akhmetshina A, Dees C, Kireva T, Gelse K, et al (2012) β-catenin is a central mediator of pro-fibrotic Wnt signaling in systemic sclerosis. Annals of the Rheumatic Diseases 71: 761–767. 10.1136/annrheumdis-2011-200568 22328737 PMC3951949

[10] Zeng G, Awan F, Otruba W, Muller P, Apte U, Tan X, et al (2007) Wnt'er in liver: Expression of Wnt and frizzled genes in mouse. Hepatology 45: 195–204. 17187422 10.1002/hep.21473

[11] Cheng JH, She H, Han YP, Wang J, Xiong S, Asahina K, et al (2007) Wnt antagonism inhibits hepatic stellate cell activation and liver fibrosis. American Journal of Physiology—Gastrointestinal and Liver Physiology 294: G39–G49. 18006602 10.1152/ajpgi.00263.2007

[12] Jiang F, Parsons CJ, Stefanovic B (2006) Gene expression profile of quiescent and activated rat hepatic stellate cells implicates Wnt signaling pathway in activation. Journal of Hepatology 45: 401–409. 16780995 10.1016/j.jhep.2006.03.016

[13] Kordes C, Sawitza I, HÃ¤ussinger D (2008) Canonical Wnt signaling maintains the quiescent stage of hepatic stellate cells. Biochemical and Biophysical Research Communications 367: 116–123. 18158920 10.1016/j.bbrc.2007.12.085

[14] Rashid ST, Humphries JD, Byron A, Dhar A, Askari JA, Selley JN, et al (2012) Proteomic analysis of extracellular matrix from the hepatic stellate cell line LX-2 identifies CYR61 and Wnt-5a as novel constituents of fibrotic liver. Journal of Proteome Research 11: 4052–4064. 10.1021/pr3000927 22694338 PMC3411196

[15] Gordon MD, Nusse R (2006) Wnt signaling: Multiple pathways, multiple receptors, and multiple transcription factors. Journal of Biological Chemistry 281: 22429–22433. 16793760 10.1074/jbc.R600015200

[16] Park JR, Jung JW, Lee YS, Kang KS (2008) The roles of Wnt antagonists Dkk1 and sFRP4 during adipogenesis of human adipose tissue-derived mesenchymal stem cells. Cell Prolif 41: 859–874. 10.1111/j.1365-2184.2008.00565.x 19040566 PMC6495667

[17] Jia L, Miao C, Cao Y, Duan EK (2008) Effects of Wnt proteins on cell proliferation and apoptosis in HEK293 cells. Cell Biol Int 32: 807–813. 10.1016/j.cellbi.2008.03.011 18462958

[18] de la Roche M, Worm J, Bienz M (2008) The function of BCL9 in Wnt/β-catenin signaling and colorectal cancer cells. BMC Cancer 8. 10.1186/1471-2407-8-199 PMC2478683 18627596

[19] Townsley FM, Cliffe A, Bienz M (2004) Pygopus and Legless target Armadillo/β-catenin to the nucleus to enable its transcriptional co-activator function. Nature Cell Biology 6: 626–633. 15208637 10.1038/ncb1141

[20] Arce L, Yokoyama NN, Waterman ML (2006) Diversity of LEF//TCF action in development and disease. Oncogene 25: 7492–7504. 17143293 10.1038/sj.onc.1210056

[21] Solberg N, MacHon O, MacHonova O, Krauss S (2012) Mouse Tcf3 represses canonical Wnt signaling by either competing for β-catenin binding or through occupation of DNA-binding sites. Molecular and Cellular Biochemistry 365: 53–63. 10.1007/s11010-012-1243-9 22270545

[22] Wu CI, Hoffman JA, Shy BR, Ford EM, Fuchs E, Nguyen H, et al (2012) Function of Wnt/β-catenin in counteracting Tcf3 repression through the Tcf3-β-catenin interaction. Development (Cambridge) 139: 2118–2129. 10.1242/dev.076067 PMC3357906 22573616

[23] Boutros M, Paricio N, Strutt DI, Mlodzik M (1998) Dishevelled activates JNK and discriminates between JNK pathways in planar polarity and wingless signaling. Cell 94: 109–118. 9674432 10.1016/s0092-8674(00)81226-x

[24] Kohn AD, Moon RT (2005) Wnt and calcium signaling: β-Catenin-independent pathways. Cell Calcium 38: 439–446. 16099039 10.1016/j.ceca.2005.06.022

[25] Li L, Yuan H, Xie W, Mao J, Caruso AM, McMahon A, et al (1999) Dishevelled proteins lead to two signaling pathways: Regulation of LEF- 1 and c-Jun N-terminal kinase in mammalian cells. Journal of Biological Chemistry 274: 129–134. 9867820 10.1074/jbc.274.1.129

[26] Bryja V, Schulte G, Rawal N, Grahn A, Arenas E (2007) Wnt-5a induces dishevelled phosphorylation and dopaminergic differentiation via a CK1-dependent mechanism. Journal of Cell Science 120: 586–595. 17244647 10.1242/jcs.03368

[27] Biechele S, Cox BJ, Rossant J (2011) Porcupine homolog is required for canonical Wnt signaling and gastrulation in mouse embryos. Developmental Biology 355: 275–285. 10.1016/j.ydbio.2011.04.029 21554866

[28] Zhang Y, Appleton BA, Wiesmann C, Lau T, Costa M, Hannoush RN, et al (2009) Inhibition of Wnt signaling by Dishevelled PDZ peptides. Nat Chem Biol 5: 217–219. 10.1038/nchembio.152 19252499

[29] Eguchi M, Nguyen C, Lee SC, Kahn M (2005) ICG-001, a novel small molecule regulator of TCF/beta-catenin transcription. Med Chem 1: 467–472. 16787331 10.2174/1573406054864098

[30] Gonzalez-Sancho JM, Greer YE, Abrahams CL, Takigawa Y, Baljinnyam B, Lee KH, et al (2013) Functional consequences of Wnt-induced dishevelled 2 phosphorylation in canonical and noncanonical Wnt signaling. J Biol Chem 288: 9428–9437. 10.1074/jbc.M112.448480 23396967 PMC3611012

[31] Christodoulides C, Lagathu C, Sethi JK, Vidal-Puig A (2009) Adipogenesis and WNT signalling. Trends in Endocrinology & Metabolism 20: 16–24. 19008118 10.1016/j.tem.2008.09.002 PMC4304002

[32] Tian X, Zhang J, Tan TK, Lyons JG, Zhao H, Niu B, et al (2012) Association of β-catenin with P-Smad3 but not LEF-1 differentiates in vitro profibrotic and anti-inflammatory effects of TGF-β1. Journal of Cell Science. 10.1242/jcs.103036 23203799

[33] Zhou B, Liu Y, Kahn M, Ann DK, Han A, Wang H, et al (2012) Interactions Between β-Catenin and Transforming Growth Factor-β Signaling Pathways Mediate Epithelial-Mesenchymal Transition and Are Dependent on the Transcriptional Co-activator cAMP-response Element-binding Protein (CREB)-binding Protein (CBP). Journal of Biological Chemistry 287: 7026–7038. 10.1074/jbc.M111.276311 22241478 PMC3293544

[34] Vuga LJ, Ben-Yehudah A, Kovkarova-Naumovski E, Oriss T, Gibson KF, Feghali-Bostwick C, et al (2009) WNT5A is a regulator of fibroblast proliferation and resistance to apoptosis. American Journal of Respiratory Cell and Molecular Biology 41: 583–589. 10.1165/rcmb.2008-0201OC 19251946 PMC2778165

[35] Griesmann H, Ripka S, Pralle M, Ellenrieder V, Baumgart S, Buchholz M, et al (2013) WNT5A-NFAT signaling mediates resistance to apoptosis in pancreatic cancer. Neoplasia 15: 11–22. 23359789 10.1593/neo.121312 PMC3556935

[36] Kim J, Kim DW, Chang W, Choe J, Kim J, Park CS, et al (2012) Wnt5a is secreted by follicular dendritic cells to protect germinal center B cells via Wnt/Ca2+/NFAT/NF-kappaB-B cell lymphoma 6 signaling. J Immunol 188: 182–189. 10.4049/jimmunol.1102297 22124122

[37] Elsharkawy AM, Oakley F, Mann DA (2005) The role and regulation of hepatic stellate cell apoptosis in reversal of liver fibrosis. Apoptosis 10: 927–939. 16151628 10.1007/s10495-005-1055-4

[38] Naskar D, Maiti G, Chakraborty A, Roy A, Chattopadhyay D, Sen M (2014) Wnt5a-Rac1-NF-kappaB homeostatic circuitry sustains innate immune functions in macrophages. J Immunol 192: 4386–4397. 10.4049/jimmunol.1302817 24706725

[39] Marra F, Romanelli RG, Giannini C, Failli P, Pastacaldi S, Arrighi MC, et al (1999) Monocyte chemotactic protein-1 as a chemoattractant for human hepatic stellate cells. Hepatology 29: 140–148. 9862860 10.1002/hep.510290107

[40] Mann J, Chu DC, Maxwell A, Oakley F, Zhu NL, Tsukamoto H, et al (2010) MeCP2 controls an epigenetic pathway that promotes myofibroblast transdifferentiation and fibrosis. Gastroenterology 138: 705–714, 714 e701–704. 10.1053/j.gastro.2009.10.002 19843474 PMC2819585

[41] Ebrahimkhani MR, Oakley F, Murphy LB, Mann J, Moles A, Perugorria MJ, et al (2011) Stimulating healthy tissue regeneration by targeting the 5-HT2B receptor in chronic liver disease. Nat Med 17: 1668–1673. 10.1038/nm.2490 22120177 PMC3428919

[42] Orford K, Crockett C, Jensen JP, Weissman AM, Byers SW (1997) Serine phosphorylation-regulated ubiquitination and degradation of Î²- catenin. Journal of Biological Chemistry 272: 24735–24738. 9312064 10.1074/jbc.272.40.24735

