# Supplementary figures and images for: Non-Canonical Wnt Predominates in Activated Rat Hepatic Stellate Cells, Influencing HSC Survival and Paracrine Stimulation of Kupffer Cells
Source: PLoS One. 2015 Nov 13;10(11):e0142794. doi: 10.1371/journal.pone.0142794 (PMC4643911; doi:10.1371/journal.pone.0142794)

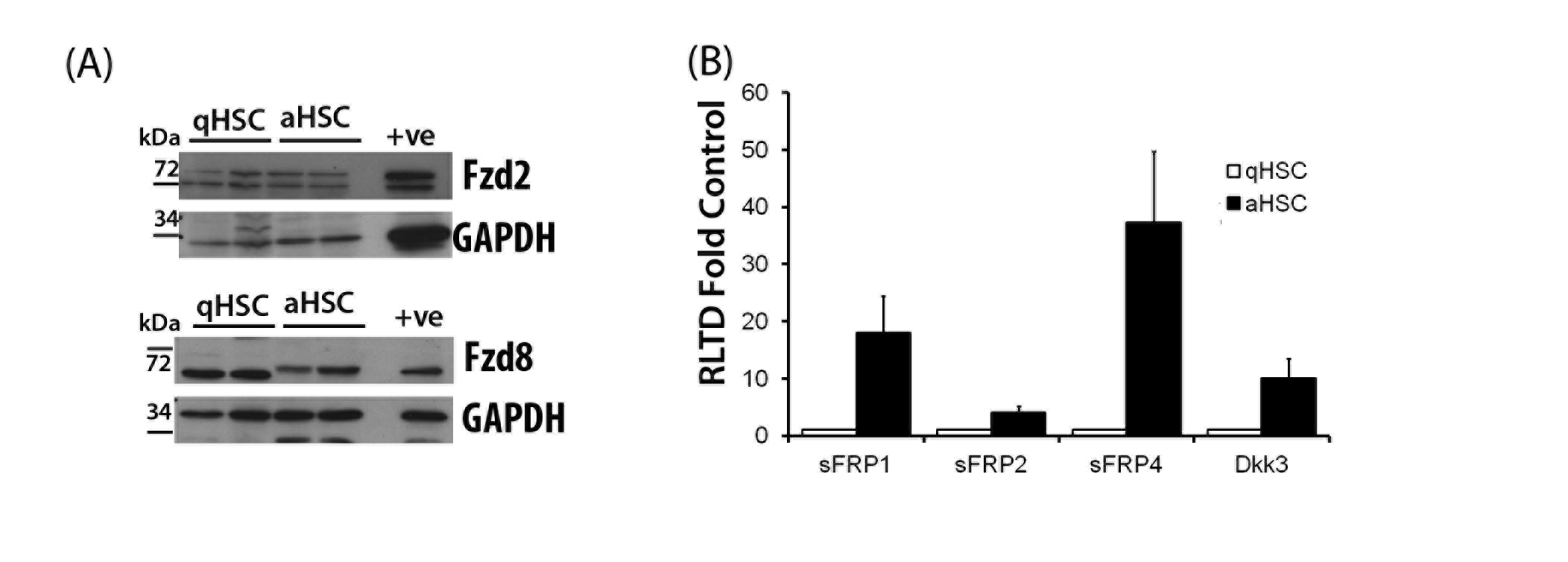

Supplement: S1 Fig — (A) Western Blot analysis confirms protein expression of Fzd2 and FZd8 in qHSC and aHSC. Fzd overexpessing LX2 cells serve as a positive control. (B) qRT-PCR analysis of sFRPs and Dkk 3 in rat qHSC and aHSC. Results expressed as fold change normalised to control ± SEM (n = 4). (TIF) [file pone.0142794.s001.tif]

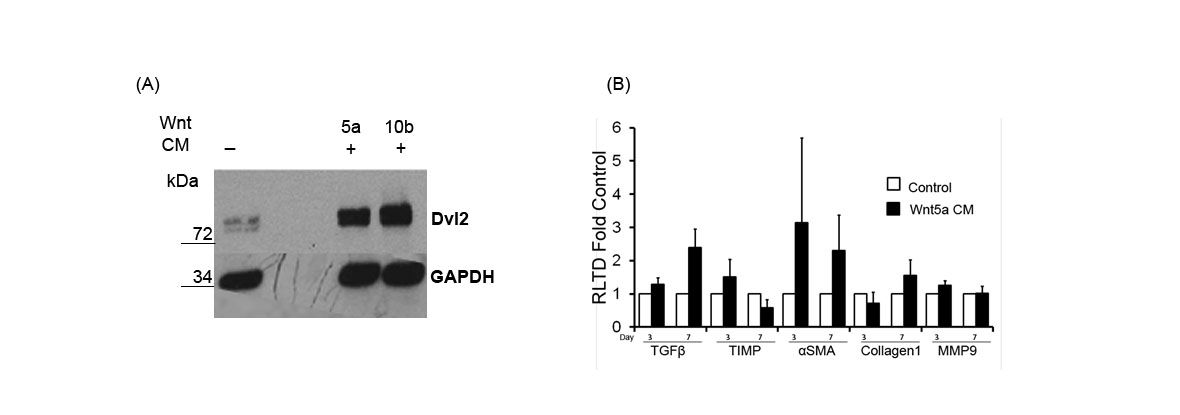

Supplement: S6 Fig — (A) Western Blot demonstrating increased expression of Dvl2 in day 7 rat HSCs upon Wnt5a or Wnt10b conditioned medium treatement (B) qRT-PCR for profibrotic markers in rat HSCs treated with Wnt5a conditioned medium at Day3 or Day 7 of in vitro culture. Results expressed as fold change normalised to control ± SEM (n = 3). (TIF) [file pone.0142794.s006.tif]
