# Supplementary material for: Non-Canonical Wnt Predominates in Activated Rat Hepatic Stellate Cells, Influencing HSC Survival and Paracrine Stimulation of Kupffer Cells
Source: PLoS One. 2015 Nov 13;10(11):e0142794. doi: 10.1371/journal.pone.0142794 (PMC4643911; doi:10.1371/journal.pone.0142794)
Supplement: S1 Table — (DOCX) [file pone.0142794.s010.docx]

| **Gene** | **Sequence (5’-3’)** | **Annealing Temp** |
| --- | --- | --- |
| hAdipsin F | CACCATCGACCACGACCTC | 60 |
| hAdipsin R | CACGTCGCAGAGAGTTCCC |  |
| hAxin2 f | CGAGATCCAGTCGGTGATGG | 58 |
| hAxin2 r | CCACACACGACCTTTAGGCT |  |
| hBCL92 F | TCTGGGCTGAGGGAGGTGGAC | 60 |
| hBCL92 R | TCTGCTGCTGCGGGGTCATCT |  |
| hCCND1 F | CCAGAGGCGGAGGAGAACAAA | 58 |
| hCCND1 R | AGCGTGTGAGGCGGTAGTAGG |  |
| hCD36 F | CGCTGAGGACAACACAGTCT | 60 |
| hCD36 R | CTGCCACAGCCAGATTGAGA |  |
| hCEBPα F | GGTGGACAAGAACAGCAACGA | 60 |
| hCEBPα R | GTCATTGTCACTGGTCAGCTC |  |
| hChibby F | GGTTAGTGGCGGTGTGGAC | 58 |
| hChibby R | AAGTGGGATTCAGCAGTGG |  |
| hcMyc F | CCACAGCAAACCTCCACACA | 58 |
| hcMyc R | ACCCTGCCACTGTCCAACTT |  |
| hCollagen1 F | CAAGAGGAAGGCCAAGTCGAGG | 58 |
| hCollagen1 R | CGTTGTCGCAGACGCAGAT |  |
| hGAPDHf | GTCAGTGGTGGACCTGACCT | 58 |
| hGAPDHr | TGAGCTTGACAAAGTGGTCG |  |
| hLRXα F | ACAAAAGCGGAAAAAGGGGC | 60 |
| hLRXα R | AAGAATCCCTTGCAGCCCTC |  |
| hPPARγ F | GGGATCAGCTCCGTGGA | 60 |
| hPPARγ R | TGCACTTTGGTACTCTTGAAGTT |  |
| hPygopus F | GTGGACTGGATGGGTTAGGAG | 58 |
| hPygopus R | GATTCGGTGGTGGAGCATACT |  |
| hPygopus2 F | GTGAACGATGACCAGGATG | 58 |
| hPygopus2 R | TGCCCTCACGGATGTAGAC |  |
| hrBCL9 F | GGTCCGTCCCCCTACAGTGAT | 60 |
| hrBCL9 R | CATTTCCAGCCCCATTCTTCA |  |
| hTGFβ R | CGCACGCAGCAGTTCTTCTCC |  |
| hTGFβ1 F | TGACAGCAGGGATAACACACT | 58 |
| hTIMP1 F | ACTTCCACAGGTCCACAAC | 58 |
| hTIMP1 R | CATTCCTCACAGCCAACAGT |  |
| hTNC F | GGTTGCTGGAGACTGTGGAA | 60 |
| hTNC R | AGGTTTTCCAGAAGGGGCAG |  |
| hWnt10b F | ACACTTGCATTTCCGCTTCAG | 58 |
| hWnt10b R | GACACTTGCATTTCCGCTTCAG |  |
| hWnt3a F | GGCTGGAAGTGGGGTGGCTGTA | 58 |
| hWnt3a R | CCTCGTTGTTGTGGCGGTTCAT |  |
| hαSMAf | CACTGTTCTCTGTGCCTAAAC | 58 |
| hαSMAr | GGTTGGCCTTTCCCAGTG |  |
| rAxin2 F | TCCTTACCGCATGGGGAGTA | 58 |
| rAxin2 R | GTGGGTTCTCGGGAAGTGAG |  |
| rCollagen1 F | TTCACCTACAGCACGCTTGTG | 58 |
| rCollagen1 R | GATGACTGTCTTGCCCCAAGTT |  |
| rEVI F | ATTGGGTCAGTGGCTCATAAG | 58 |
| rEVI R | GATGAAGATGCTGGGTGTGAG |  |
| rFzd1 F | GCGACGTACTGAGCGGAGTG | 58 |
| rFzd1 R | TGATGGTGCGGATGCGGAAG |  |
| rFzd2 F | CTCAAGGTGCCGTCCTATCTCAG | 58 |
| rFzd2 R | GCAGCACAACACCGACCATG |  |
| rFzd4 F | CCCCACAAAACTCCCATCCAGC | 55 |
| rFzd4 R | CCACACGGCCATCCAAATATCC |  |
| rFzd5 F | CTGGAGTCTTGGCGGCGCTTCA | 58 |
| rFzd5 R | GCCCGGTCCTGCCAGTGAGTGC |  |
| rFzd6 F | AGACCTCCCCAGAAGCATCCCT | 58 |
| rFzd6 R | TTCTCCTTCACGGTGCCTGCTC |  |
| rFzd8 F | GCGAGTACGAGGAGCTGGGTGC | 58 |
| rFzd8 R | CCGCCAAGAACCACGTGAGAGA |  |
| rhWnt5a F | AACCCTGTTCAGATGTCAGAAG | 58 |
| rhWnt5a R | CTGCATGTGGTCCTGATACAAG |  |
| rNFATc3 F | GAGAAGAGTCCTGGCATCAAC | 58 |
| rNFATc3 R | GAGTGATGGGGTGAAAGAGAG |  |
| rPPARγ F | ATTTCTGCTCCACACTAT | 58 |
| rPPARγ R | GCTTTATCCCCACAGACT |  |
| rPTK7 F | CCCCTCAGCACCAAACAGAAG | 58 |
| rPTK7 R | GGACACCTTCACCTGCCTCTG |  |
| rROR1 F | CAGGGGGAAATAGAAAATCAG | 58 |
| rROR1 R | ACAGTATGGGAAGGCGTAGTG |  |
| rROR2 F | CGTGCGGTGGCTGAAGAATG | 60 |
| rROR2 R | GCCCGTGTCTGTCGTGTCCA |  |
| rRYK F | TCCACCCAGACGACCCAGTAT | 58 |
| rRYK R | CTTCACCTTGGCTTTGGCTTC |  |
| rSfrp1 F | GCTCAACAAGAA CTGCCACATG | 58 |
| rSfrp1 R | CCAGTAGAAGCCGAAGAACTGC |  |
| rSfrp2 F | CCTGGAGACAAAGAGCAAGACC | 58 |
| rSfrp2 R | TGACCAGATAGGGAGCGT TGAT |  |
| rSfrp4 F | CTTCCCACTGTGTGGACCTT | 65 |
| rSfrp4R | CTATCCCTCGAACGCAAGTC |  |
| rSfrp5 F | GAGATGCTGCACTGCCACAAGT | 60 |
| rSfrp5 R | ACGAAGTCGCTGGAGCACATCT |  |
| rSOX9 F | TGGCAGAGGGTGGCAGACAGC | 58 |
| rSOX9 R | TTGGGCGGCAGGTATTGGTCA |  |
| rTGFβ F | GAACCCCCATTGCTGTCC | 58 |
| rTGFβ R | TCCGTCTCCTTGGTTCAG |  |
| rTIMP1 F | GCATGGACATTTATTCTCCAC | 58 |
| rTIMP1 R | TCTCTAGGAGCCCCGATCTG |  |
| rWnt3a F | TGGGTTGTCTGGCAGGTGTGAG | 58 |
| rWnt3a R | TCC ACCACCATCTCTGAGGCAC |  |
| rWnt4 F | CTGGAGAAGTGTGGCTGTGACC | 58 |
| rWnt4 R | AAGGACTGTGAGAAGGCTACGC |  |
| rWnt6 F | GGGAGGCTGCGGAGATGATGTC | 58 |
| rWnt6 R | CAGAGCACAGGAACCCGAAAG C |  |
| rWnt7b F | GGACGGCTGTGACACCATGTGC | 58 |
| rWnt7b R | GGTGAAGACCTCGGTGCGCTCG |  |
| rWnt9b F | CGGCCCAGCAAGTACTCACCTG | 58 |
| rWnt9b R | GCACTCCACGTAGCAGCACCAC |  |
| rαSMA F | CATGTCGTCCCAGTTGGTGAT | 58 |
| rαSMA R | CGAAGCGCAGAGCAAGAGA |  |
| rβActin F | AGAGGGAAATCGTGCGTGACA | 58 |
| rβActin R | ACATCTGCTGGAAGGTGGACA |  |
